# High-resolution taxonomic examination of the oral microbiome after oil pulling with standardized sunflower seed oil and healthy participants: a pilot study

**DOI:** 10.1007/s00784-020-03582-0

**Published:** 2020-09-19

**Authors:** Tim Griessl, Silke Zechel-Gran, Stefan Olejniczak, Markus Weigel, Torsten Hain, Eugen Domann

**Affiliations:** Institute of Medical Microbiology, German Center for Infection Research (DZIF Partner Site Giessen-Marburg-Langen), Justus-Liebig-University Giessen, Schubertstrasse 81, 35392 Giessen, Germany

**Keywords:** Oil pulling, Sunflower oil, Oral microbiome, Inflammation, Infection, Biofilm

## Abstract

**Objectives:**

We aimed at the high-resolution examination of the oral microbiome depending on oil pulling, compared it with saline pulling, and analyzed whether the method is capable of reducing the overall microbial burden of the oral cavity.

**Materials and methods:**

The study was a cohort study with three healthy subjects. Oil pulling samples, saline pulling samples, and saliva samples were microscoped and cultured under microaerophilic and anaerobic conditions; colony-forming units were counted; and cultivated bacteria were identified employing MALDI-TOF MS. The oral microbiomes (saliva) and the microbiota incorporated in oil and saline pulling samples were determined in toto by using 16S rDNA next-generation sequencing (NGS) and bioinformatics.

**Results:**

Microscopy revealed that oral epithelial cells are ensheathed with distinct oil droplets during oil pulling. Oil pulling induced a higher production of saliva and the oil/saliva emulsion contained more bacteria than saline pulling samples. Oil pulling resulted in a significant and transient reduction of the overall microbial burden in comparison to saliva examined prior to and after pulling. Both oil and saline pulling samples mirrored the individual oral microbiomes in saliva.

**Conclusions:**

Within the limitations of this pilot study, it might be concluded that oil pulling is able to reduce the overall microbial burden of the oral cavity transiently and the microbiota in oil pulling samples are representative to the oral microbiome.

**Clinical relevance:**

Within the limitations of this pilot study, it might be concluded that oil pulling can be considered as an enlargement of standard oral hygiene techniques since it has the characteristic of an oral massage, enwrapping epithelial cells carrying bacteria in oil vesicles and reaching almost all unique habitats in oral cavity.

## Introduction

The oral cavity is a very complex space of the human body providing unique habitats for microbial colonization comprising buccal and vestibular mucosa, lips, cheek, palate, tongue, and natural (teeth) and artificial solid surfaces (dental materials). Saliva baths the oral tissues and fluctuations in oral parameters such as temperature, oxygen availability, pH, and variability in the composition and frequency of exposure to dietary constituents occur and result in a highly diversified oral environment. The characteristic of the oral cavity is the special balanced contention between bacterial colonization and the often broken barrier between bone and environment (periodontitis, dental implants, traumata) both by the influence of the immune system and saliva which contains IgA, lactoferrin, lysozyme, growth factors, and cellular defense mechanisms [1–3].

Although the oral microbiome is in principle composed of bacteria, viruses, fungi, archaea, and protozoa, the predominant microorganisms are bacteria. Members of the bacterial microbiota are mainly responsible for local and distant-site infections, in particular in the case of poor oral hygiene. Local infections encompass caries, gingivitis, and periodontitis [4, 5]. Distant-site infections can appear as acute infections such as bacteremia or even sepsis [6], as infective endocarditis [7], idiopathic arthritis [8], atherosclerosis and chronic inflammation [9, 10], and stroke [11]. Additionally, women with periodontitis show a higher prevalence of preterm low birth weight infants [9, 12].

In order to reduce the microbial burden of the oral cavity and to avoid or to minimize the abovementioned infectious diseases, people are engaged in regular oral hygiene practices, typically consisting of brushing with toothpastes, utilizing dental floss, and/or rinsing with mouthwashes. A crucial point is the capacity of bacteria to form polymicrobial biofilms since their removal requires typically mechanical techniques and preventive strategies to limit oral dysbiosis [1, 3, 13, 14].

“Oil pulling” or “oil swishing” is part of Ayurveda, a holistic system of medicine, which evolved in India and which is now practiced in other parts of the world as a form of complementary and alternative medicine. It is a procedure that involves swishing oil (in general sunflower or sesame oil, rarely coconut oil) in the mouth for oral and systemic health benefits. Occasionally, these oils are enriched with natural products such as herbs or etherous ingredients and are not standardized. Oil pulling has been used to prevent tooth decay, oral malodor, bleeding gums, dryness of throat, cracked lips, and for strengthening teeth, gums, and the jaw. In particular in cases of mouth ulcer or oral cancer, where teeth’s brushing is impeded, oil pulling can be used to clean the oral cavity. Therefore, it is a specialized technique to treat as well as to prevent oral diseases [15, 16]. Miscellaneous studies, including randomized clinical trials, have been performed counting bacterial colonies, calculating the amount of *Streptococcus mutans*, and assessing plaque and gingival index [17–22], but the studies were not standardized with regard to oil, volume, and duration of pulling. Therefore, the results are still controversial.

We aimed at the high-resolution taxonomic examination of the oral microbiome prior to and after oil pulling and compared it with saline pulling. Furthermore, we analyzed whether oil and saline pulling are able to reduce the overall microbial burden of the oral cavity and determined the appropriate volume of oil and duration for oil pulling.

## Materials and methods

### Subjects

The study was done with the encouragement of three healthy subjects, all nonsmoker and omnivore: subject A: male; subject B: male; subject C: female. They were Germans and local residents. All of the participants were able to pull with sunflower seed oil and saline (0.9% NaCl) and were part of the laboratory team in order to ensure continuity and immediate sample processing. Written informed consent was obtained from the individuals for publication of this study and any accompanying images. The study was approved by the Ethics Board of the Justus-Liebig-University of Giessen (reference number: AZ 97/16).

### Procedure of pulling

**Fig. 1:**
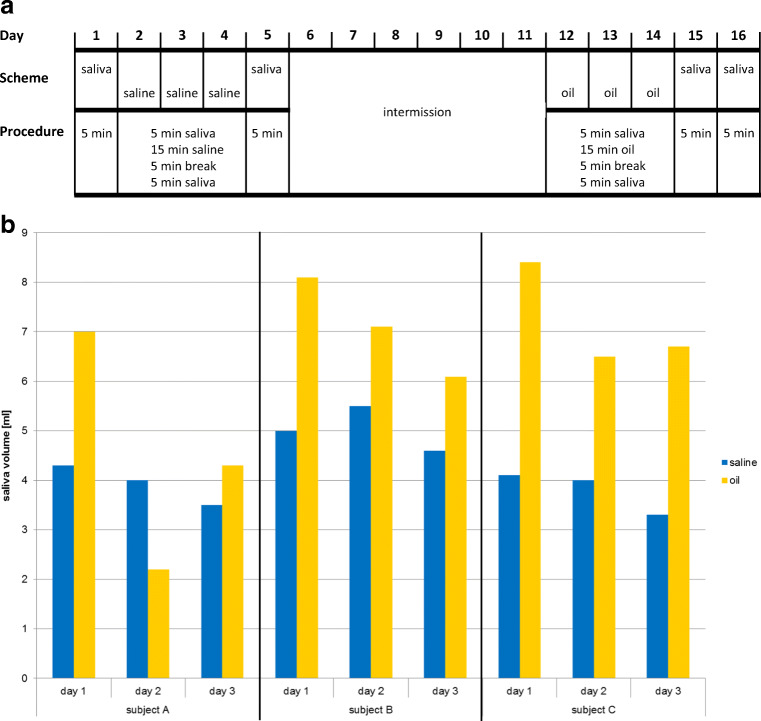
Pulling with saline and sunflower seed oil. **a** Pulling scheme. The study was separated into three parts: pulling with saline (0.9% NaCl), intermission, and pulling with oil (sunflower seed oil). **b** Saliva production due to saline pulling (blue) and oil pulling (yellow)

The study lasted 16 consecutive days and was separated into three parts (Fig. 1): pulling with saline (0.9% NaCl = negative control) for 3 days, intermission of 6 days, pulling with oil (sunflower seed oil) for 3 days. The pulling substances had a volume of 15 ml and were provided in 50-ml tubes (Eppendorf, Hamburg, Germany). The pulling lasted 15 min and was done in the morning between 6 and 7 o’clock: after getting up and before oral hygiene and/or breakfast. After 15 min of pulling, the mixture of saline/saliva and the mixture of oil/saliva were collected in the same tube, respectively. Prior to and 5 min after pulling, the saliva produced within 5 min was collected in separate 50-ml tubes (Eppendorf, Hamburg, Germany). In total, 22 samples were collected for each subject (Table 1), altogether 66 for the three subjects.

**Table 1:** Overview of the samples (*n* = 22) collected for analysis per subject

Data point no.	Day	Sample
01	01	Saliva
02	02	Saliva prior to pulling
03	02	Saline pulling (day 1)
04	02	Saliva after pulling
05	03	Saliva prior to pulling
06	03	Saline pulling (day 2)
07	03	Saliva after pulling
08	04	Saliva prior to pulling
09	04	Saline pulling (day 3)
10	04	Saliva after pulling
11	05	Saliva
12	12	Saliva prior to pulling
13	12	Oil pulling (day 1)
14	12	Saliva after pulling
15	13	Saliva prior to pulling
16	13	Oil pulling (day 2)
17	13	Saliva after pulling
18	14	Saliva prior to pulling
19	14	Oil pulling (day 3)
20	14	Saliva after pulling
21	15	Saliva
22	16	Saliva

### Sunflower seed oil used

The sunflower seed oil *Oleum Helianthi raffinatum* (article number 7309) distributed by company Caelo (Hilden, Germany) under CAS number 8001-21-6 and EG number 232-273-9 was used. It is a concoction of 16:0 palmitic acid (4–9%), 18:0 stearic acid (1–7%), 18:1 oleic acid (14–40%), and 18:2 linoleic acid (48–74%). This commercially available oil is standardized; free of herbs, ethereous, or other ingredients; and certified. Standardization and commercial availability are important in order to compare studies.

### Staining and microscopy

For microscopic analysis, samples of saliva, saline, and oil pulling were stained with Giemsa (Merck, Darmstadt, Germany). Therefore, the samples were diluted 1:4 (50 μl Giemsa stain:150 μl sample) and 20 μl of each dilution was transferred on a slide and covered with a cover slip. The samples were examined with the Biozero BZ 8000 microscope (Keyence, Neu-Isenburg, Germany) and recorded.

### Bacterial culture and identification by MALDI-TOF MS

One-hundred microliter of each sample was streaked on sheep blood, MacConkey, chocolate, Sabouraud, and Schaedler agar plates. The latter ones were incubated anaerobically at 37 °C for 5 days, the others in 5% CO_2_ atmosphere at 37 °C for 48 h. In order to determine the amount of bacteria in each sample, serial dilutions were done with 0.9% NaCl, plated on sheep blood agar plates, and incubated at 37 °C and 5% CO_2_ for 48 h. Colony-forming units (CFU) were counted and bacteria were identified by using MALDI-TOF MS: matrix-assisted laser desorption ionization time of flight mass spectrometry [23].

### Nucleic acid extraction of samples, library construction, and 16S rDNA next-generation sequencing

For nucleic acid extraction, the following samples have been used: saliva (1.5 ml), saline/saliva mixture (1.5 ml), oil/saliva mixture (1.5 ml), saline/mock community mixture (1.3 ml/0.2 ml = 1.5 ml), oil/mock community mixture (1.3 ml/0.2 ml = 1.5 ml). In order to examine the potential bacterial background in the solutions used, negative controls of water (1.5 ml), saline (1.5 ml), and oil (1.5 ml) have been used.

Samples were centrifuged in 1.5-ml Eppendorf tubes (13,000 rpm, 10 min) and DNA from the bacterial pellets was extracted by using glass beads and the Power Lyzer DNA Isolation Kit from MoBio as recommended by the vendor (MoBio Laboratories, Carlsbad, CA, USA). The supernatants from the samples containing oil appeared as an emulsion of an oleaginous and an aqueous phase. These mixtures were treated with a solution of phenol/chloroform:isoamyl ethanol to remove oleaginous phase and DNA was precipitated with salt and ethanol. DNA of all samples was resuspended in 100 μl of nuclease-free water and concentration was determined using Qubit Fluorometric Quantitation (Thermo Fisher Scientific, Waltham, MA, USA). In order to examine the entire oil/saliva samples, DNA from pellets and DNA from supernatants were mixed in proportions of 4:1 and similar amounts of saline/saliva samples with water (4:1) to achieve the same dilution factor. The V4 region of 16S rRNA gene was amplified using adapter forward primer 5′-TCGTCGGCAGCGTCAGATGTGTATAAGAGACAGGTGCCAGCMGCCGCGGTAA-3′, adapter reverse primer 5′-GTCTCGTGGGCTCGGAGATGTGTATAAGAGACAGGGACTACHVGGGTWTCTAAT-3′, and the 2× Kapa HiFi HotStart Ready Mix (Kapa Biosystems, Wilmington, MA, USA). Amplification profile comprised an initial heating step at 95 °C for 3 min, 25 cycles of denaturation at 95 °C for 30 s, annealing at 55 °C for 30 s, elongation at 72 °C for 30 s, and a final elongation step at 72 °C for 5 min. PCR products were purified with Agencourt AMPure XP system as recommended by the vendor (Beckman Coulter, Brea, CA, USA). Size, purity, and concentration of amplicons were determined using the Agilent Bioanalyzer as recommended by the vendor (Agilent Technologies, Santa Clara, CA, USA). The index PCR was done by using the Nextera index Kit v2 Set B as recommended by the vendor (Illumina, San Diego, CA, USA). The quality of the index PCR has been determined as described above for the adapter PCR. The library was adjusted to 3 pM, the flow cell was prepared and loaded according to the Reagent Preparation Guide of MiSeq Reagent Kit v2 as recommended by the vendor (Illumina), and the MiSeq sequencing machine was started for sequencing.

### Mock community

The mock community served as the positive control and was a composition of 14 Gram-positive and Gram-negative rods and cocci, considering morphologies and structure of cell walls (Table 2). The bacteria were cultured in appropriate media and each strain had a final concentration of ~ 1 × 10^8^/ml, altogether ~ 1–2 × 10^9^/ml.

**Table 2:** Mock community

No.	Morphology	Bacterium	Strain designation
01	Gram-positive rod	*Bacillus cereus* 13	DSM 31
02	Gram-positive rod	*Lactobacillus delbrueckii* #22	DSM 103825
03	Gram-positive rod	*Listeria monocytogenes* EGDe	EDCC 2100
04	Gram-positive rod	*Propionibacterium acnes* KPA 171202	DSM 16379
05	Gram-positive coccus	*Enterococcus faecalis* Symbioflor 1	DSM 16431
06	Gram-positive coccus	*Staphylococcus aureus* DFS 42	EDCC 5430
07	Gram-positive coccus	*Streptococcus agalactiae* G 19	DSM 2134
08	Gram-positive coccus	*Streptococcus pneumoniae* TIGR 4	EDCC 5501
09	Gram-negative rod	*Acinetobacter baumannii* 65	EDCC 5502
10	Gram-negative rod	*Escherichia coli* MG 1655	DSM 18039
11	Gram-negative rod	*Porphyromonas gingivalis* W 83	EDCC 5503
12	Gram-negative rod	*Pseudomonas aeruginosa* PAO1	DSM 19880
13	Gram-negative coccus	*Moraxella catarrhalis*	DSM 9143
14	Gram-negative coccus	*Neisseria meningitidis* 21	DSM 10036

*DSM* German collection of microorganisms, *EDCC* Eugen Domann Culture Collection

### Bioinformatics

MiSeq reporter software was used to split the sequences by barcode and to generate the fastq files [24]. After quality control with FastQC [25], paired end reads were joined and primer sequences were removed with PANDAseq [26]. Filtering was done for the calculated amplicon length and reads with ambiguous base calls or with homopolymers longer than eight nucleotides were removed. Microbiomic analysis was done by using QIIME [27]. The operational taxonomic units (OTU) were analyzed with uclust [28] using the Greengenes database [29] as reference with a similarity of 97%. For each OTU, a representative sequence was chosen and aligned with PyNAST [30] using the Greengenes core reference alignment [29]. Chimeric sequences were removed by using ChimeraSlayer [31]. Taxonomic assignment was done with the uclust consensus taxonomy assigner and the Greengenes database as taxonomy reference [32]. A phylogenetic tree was created using FastTree [33]. Alpha and beta diversity analysis and taxa summary plots were generated using QIIME core diversity analysis script. The principal coordinates analysis (PCoA) plots were visualized using EMPeror [34].

### Statistical analysis

Statistical analysis has been done with MS Excel version 14.0.7177.5000.

## Results

### Oil pulling induced higher production of saliva than pulling with saline

The study lasted 16 consecutive days and was separated into three parts: pulling with saline (0.9% NaCl) for 3 days, intermission of 6 days, pulling with oil (sunflower seed oil) for 3 days (Fig. 1a; Table 1). The collected volume of saliva alone within 5 min was constantly ~ 2–3 ml. Both saline pulling and oil pulling induced production of saliva resulting in a concoction of saline/saliva and oil/saliva. The production of saliva due to oil pulling was—apart from subject A: day 2, oil pulling (Fig. 1b; sample 16 in Table 1)—always higher than due to saline pulling (Fig. 1b).

### Phenotypes of oral epithelial cells derived from saliva, saline pulling, and oil pulling samples were significantly different

**Fig. 2:**
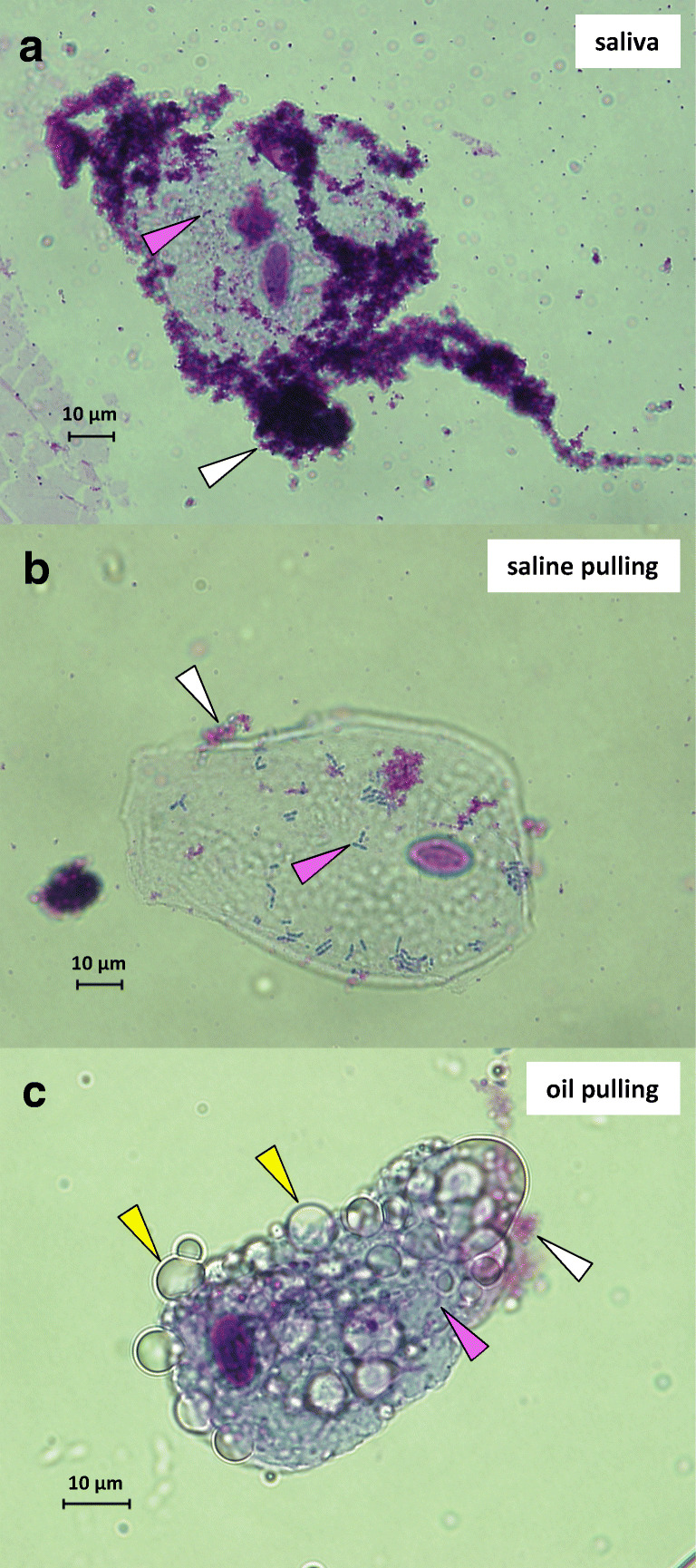
Giemsa staining and microscopy of oral epithelial cells. Cells shown are representative and derived from subject B. **a** Saliva sample. **b** Saline pulling sample. **c** Oil pulling sample. White arrow heads indicate protein aggregates, magenta arrow heads bacteria, and yellow arrow heads oil droplets. Magnification × 60; bars indicate 10 μm

Saliva, saline pulling, and oil pulling samples were stained with Giemsa and examined under a microscope. The phenotypes of oral epithelial cells derived from the three sample types were significantly different: saliva samples showed loads of protein aggregates (white arrow heads) around epithelial cells (Fig. 2a) which were barely visible in saline pulling samples (Fig. 2b) and oil pulling samples (Fig. 2c). Additionally, the latter ones showed loads of oil droplets (yellow arrow heads). Microscopy showed that all of the epithelial cells were surrounded by bacteria (magenta arrow heads). The saline/saliva samples were fluid, whereas the oil/saliva samples had the character of an emulsion.

### Oil/saliva concoction contained more bacteria than concoction of saline/saliva

**Fig. 3:**
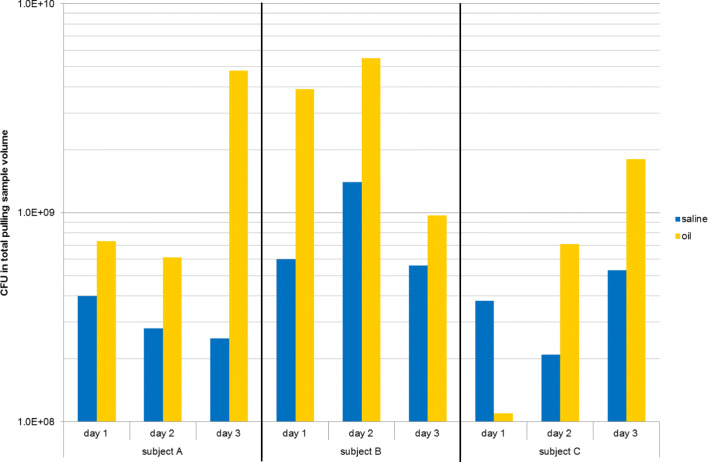
Determination of the total colony-forming units (CFU) of saline pulling samples (blue: numbers 03, 06, 09) and oil pulling samples (yellow: numbers 13, 16, 19) for subjects A, B, and C (Table 1)

Determination of the total amount of cultivable bacteria in samples of saline pulling (Fig. 3, blue bars) and oil pulling (Fig. 3, yellow bars) revealed a significant higher amount of bacteria for oil pulling (Fig. 3). One exception was a sample from subject C: day 1, oil pulling (Fig. 3; sample 13 in Table 1).

The following bacteria have been cultivated and identified by using MALDI-TOF MS (alphabetical order): *Achromobacter xylosoxidans*, *Actinomyces odontolyticus*, *Actinomyces radingae*, *Actinomyces viscosus*, *Eikenella corrodens*, *Fusobacterium nucleatum*, *Gemella sanguinis*, *Haemophilus parainfluenzae*, *Klebsiella oxytoca*, *Leifsonia aquatica*, *Leuconostoc mesenteroides*, *Micrococcus luteus*/*lylae*, *Neisseria mucosa*, *Neisseria subflava*, *Nocardia asteroides*, *Prevotella denticola*, *Prevotella desiens*, *Prevotella melaninogenica*, *Propionibacterium avidum*, *Rothia mucilaginosa*, *Serratia liquefaciens*, *Serratia marcescens*, *Staphylococcus aureus*, *Staphylococcus warneri*, *Streptococcus constellatus*, *Streptococcus mitis*/*oralis*, *Streptococcus parasanguinis*, *Streptococcus salivarius*, *Streptococcus vestibularis*, *Veillonella parvula*. Altogether, 12 out of 85 genera detected by NGS have been cultivated and seven cultivated genera (*Achromobacter*, *Klebsiella*, *Leifsonia*, *Leuconostoc*, *Micrococcus*, *Nocardia*, *Serratia*) were not detected by NGS in saliva (Table 4). *Candida albicans* was the only fungus which has been cultivated.

### Reduction of microbial burden in saliva was significantly higher for oil pulling than for pulling with saline

**Fig. 4:**
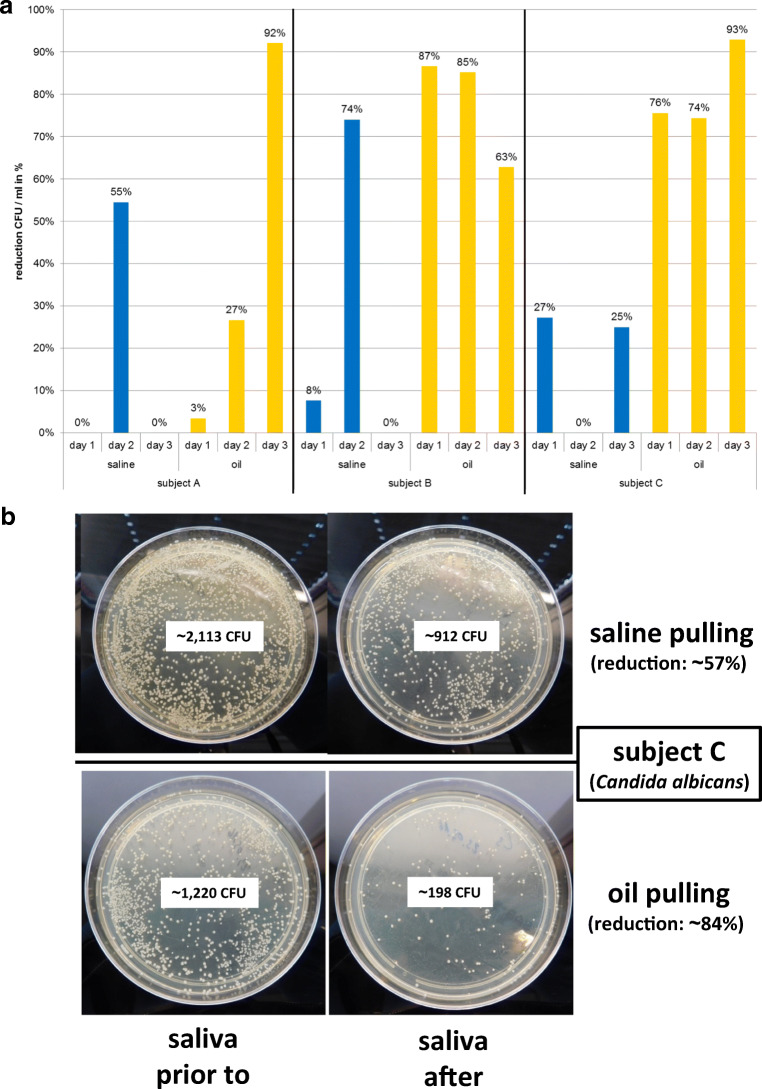
Determination of the capacity of saline pulling and oil pulling to reduce the microbial burden in saliva. Colony-forming units (CFU) of saliva prior to and after saline pulling and oil pulling were counted (Table 3). **a** Reduction of CFU per ml in percent of saliva due to saline pulling (blue bars) and oil pulling (yellow bars) of subjects A, B, and C. **b** Reduction of *Candida albicans* of subject C after saline pulling (~ 57%) and oil pulling (~ 84%) shown on Sabouraud agar plates

Prior to and after pulling with saline and oil, the colony-forming units (CFU) in saliva per milliliter were determined (Fig. 4a; Table 3). The analysis revealed a significant higher reduction of bacteria in saliva for oil pulling than for saline pulling, which was very prominent for subjects B (reduction: ~ 78.33 ± 10.87%) and C (reduction: ~ 81.00 ± 8.52%). The reduction through oil pulling for subject A increased over 3 days and achieved the same level as for subjects B and C at day 3 (Fig. 4a).

**Table 3:** Determination of the colony-forming units (CFU) per milliliter of saliva prior to and after saline pulling and oil pulling for subjects A, B, and C

Subject	Pulling with	Day	CFU/ml prior to/after	Comment
A	Saline	1	2.1 × 10^8^/4.1 × 10^8^	No reduction
		2	2.2 × 10^8^/1.0 × 10^8^	Reduction: 55%
		3	2.0 × 10^8^/5.1 × 10^8^	No reduction
	Oil	1	2.9 × 10^8^/2.8 × 10^8^	Reduction: 3%
		2	4.5 × 10^8^/3.3 × 10^8^	Reduction: 27%
		3	1.4 × 10^9^/1.1 × 10^8^	Reduction: 92%
B	Saline	1	2.6 × 10^8^/2.4 × 10^8^	Reduction: 8%
		2	8.1 × 10^8^/2.1 × 10^8^	Reduction: 74%
		3	2.3 × 10^8^/3.4 × 10^8^	No reduction
	Oil	1	6.0 × 10^8^/8.0 × 10^7^	Reduction: 87%
		2	6.0 × 10^8^/8.9 × 10^7^	Reduction: 85%
		3	4.3 × 10^8^/1.6 × 10^8^	Reduction: 63%
C	Saline	1	2.2 × 10^8^/1.6 × 10^8^	Reduction: 27%
		2	1.4 × 10^8^/1.4 × 10^8^	No reduction
		3	2.4 × 10^8^/1.8 × 10^8^	Reduction: 25%
	Oil	1	8.2 × 10^8^/2.0 × 10^8^	Reduction: 76%
		2	4.3 × 10^8^/1.1 × 10^8^	Reduction: 74%
		3	6.4 × 10^8^/4.5 × 10^7^	Reduction: 93%

Subjects A and B harbored only single colonies of *Candida* species in their oral cavities, whereas subject C harbored high amounts, which were identified as *C. albicans* by MALDI-TOF MS. Since the method used for 16S rDNA next-generation sequencing detected specifically bacteria but not fungi, reduction of this fungus due to saline pulling (~ 57%) and oil pulling (~ 84%) is representative as shown in Fig. 4b. Both methods are able to reduce the amount of *C. albicans* in the oral cavity with a significantly higher efficiency for oil pulling.

### Subjects A, B, and C harbored an individual oral microbiome

**Fig. 5:**
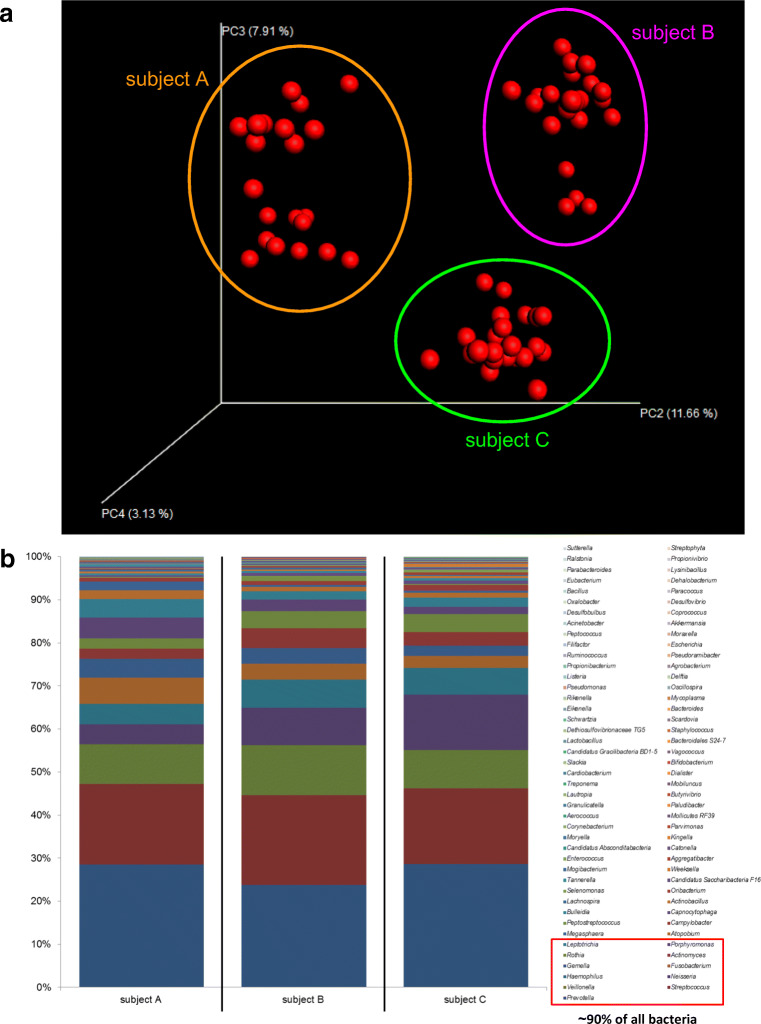
Oral microbiomes of subjects A, B, and C. **a** Principal coordinates analysis (PCoA) of *n* = 22 data points (Table 1) of subjects A, B, and C. Data points from each subject appear as distinct cloud, indicated in orange (subject A), purple (subject B), and green (subject C). **b** Percentage of bacteria down to the genus level indicated as columns for saliva of subjects A, B, and C (Table 3). Red rectangle highlights ~ 90% of all bacteria found (11 genera): *Prevotella*, *Streptococcus*, *Veillonella*, *Neisseria*, *Haemophilus*, *Fusobacterium*, *Gemella*, *Actinomyces*, *Rothia*, *Porphyromonas*, *Leptotrichia*

16S rDNA next-generation sequencing and bioinformatics revealed individual oral microbiomes for subjects A, B, and C (Fig. 5; Table 4). This was demonstrated by principal coordinates analysis (PCoA) of 22 data points comprising all, saliva, saline, and oil pulling samples (Table 1). The individual oral microbiomes appeared as distinct clouds clearly separated (Fig. 5a). Altogether, 85 genera have been detected in oral microbiome represented by saliva of probands (Fig. 5b; Table 4). Subject A harbored 75, subject B harbored 70, and subject C harbored 71 genera and they shared 60 genera. The majority of bacteria were made up of ~ 90% comprising 11 genera (decreasing order): *Prevotella*, *Streptococcus*, *Veillonella*, *Neisseria*, *Haemophilus*, *Fusobacterium*, *Gemella*, *Actinomyces*, *Rothia*, *Porphyromonas*, *Leptotrichia*. The remaining ~ 10% encompassed 74 genera.

**Table 4:** Summary of bacteria down to genus level detected in oral microbiomes of subjects A, B, and C by using 16S rDNA next-generation sequencing and bioinformatics (alphabetical order; percentage of appearance in saliva)

Genus	% of appearance in saliva			Genus	% of appearance in saliva		
	Subject A	Subject B	Subject C		Subject A	Subject B	Subject C
*Acinetobacter*	-	0.0002	0.0003	*Listeria*	0.0002	0.0012	0.0008
*Actinobacillus*	0.4555	0.3082	0.5673	*Lysinibacillus*	-	0.0002	-
*Actinomyces*	2.4590	4.7042	3.2081	*Megasphaera*	2.0961	0.4093	0.3995
*Aerococcus*	0.1050	0.0002	0.0003	*Mobiluncus*	-	0.0249	0.0620
*Aggregatibacter*	0.1753	0.1427	0.2611	*Mogibacterium*	0.3825	0.3154	0.1561
*Agrobacterium*	0.0004	0.0005	0.0005	*Mollicutes* RF39	0.0647	0.0407	0.0479
*Akkermansia*	0.0002	0.0002	0.0003	*Moraxella*	0.0004	-	0.0008
*Atopobium*	1.9965	0.9902	1.1808	*Moryella*	0.1146	0.0005	0.1020
*Bacillus*	-	0.0002	-	*Mycoplasma*	0.0011	0.0002	0.0022
*Bacteroidales* S24-7	0.0054	0.0035	0.0044	*Neisseria*	4.5475	8.6061	12.9174
*Bacteroides*	0.0018	0.0026	0.0016	*Oribacterium*	0.2185	0.3268	0.5453
*Bifidobacterium*	0.0040	-	0.0321	*Oscillospira*	0.0007	0.0007	0.0011
*Bulleidia*	0.4242	0.4873	0.6032	*Oxalobacter*	0.0002	0.0002	-
*Butyrivibrio*	-	0.0316	0.0617	*Paludibacter*	0.0992	0.0014	0.0011
*Campylobacter*	0.7986	0.9012	1.3790	*Parabacteroides*	-	0.0002	-
Candidatus *Absconditabacteria*	0.0098	0.0030	0.2660	*Paracoccus*	-	0.0005	-
Candidatus *Gracilibacteria* BD1-5	0.0154	0.0012	0.0008	*Parvimonas*	0.0271	0.1371	0.0057
Candidatus *Saccharibacteria* F16	0.2995	0.2656	0.4631	*Peptococcus*	-	0.0009	-
*Capnocytophaga*	0.3136	0.6781	0.7960	*Peptostreptococcus*	0.3143	1.2703	0.2208
*Cardiobacterium*	0.0139	0.0139	0.0041	*Porphyromonas*	4.9405	2.6363	1.6257
*Catonella*	0.1260	0.2022	0.1381	*Prevotella*	28.5108	23.7212	28.6407
*Coprococcus*	0.0004	-	-	*Propionibacterium*	0.0011	-	0.0003
*Corynebacterium*	0.0911	0.0493	0.0286	*Propionivibrio*	0.0002	-	-
*Dehalobacterium*	0.0002	-	-	*Pseudomonas*	0.0004	0.0007	0.0014
*Delftia*	0.0004	0.0007	0.0011	*Pseudoramibacter*	0.0004	-	0.0011
*Desulfobulbus*	0.0004	-	-	*Ralstonia*	0.0002	-	-
*Desulfovibrio*	0.0002	-	0.0003	*Rikenella*	0.0018	0.0009	0.0003
*Dethiosulfovibrionaceae* TG5	0.0092	-	-	*Rothia*	2.2709	3.9489	4.1385
*Dialister*	0.0470	0.0149	0.0103	*Ruminococcus*	0.0009	0.0002	0.0003
*Eikenella*	0.0004	0.0019	0.0022	*Scardovia*	0.0045	0.0016	0.0024
*Enterococcus*	0.1394	0.2259	0.2012	*Schwartzia*	0.0051	-	0.0008
*Escherichia*	0.0002	0.0002	0.0008	*Selenomonas*	0.1298	0.2466	0.6986
*Eubacterium*	0.0002	-	-	*Slackia*	0.0170	0.0012	0.0003
*Filifactor*	-	0.0007	0.0005	*Staphylococcus*	0.0058	0.0044	0.0005
*Fusobacterium*	6.0281	3.7349	2.8685	*Streptococcus*	18.6900	20.8486	17.5940
*Gemella*	4.3812	3.6017	2.3314	*Streptophyta*	-	-	0.0003
*Granulicatella*	0.0222	0.0425	0.0373	*Sutterella*	0.0002	-	-
*Haemophilus*	4.7774	6.5329	6.1481	*Tannerella*	0.5797	0.1848	0.1634
*Kingella*	0.0508	0.0600	0.1150	*Treponema*	0.0298	0.0112	0.0416
*Lachnospira*	0.4340	0.4620	0.1640	*Vagococcus*	0.0049	0.0098	0.0041
*Lactobacillus*	0.0022	0.0009	0.0098	*Veillonella*	9.3103	11.6926	8.8377
*Lautropia*	0.0537	0.0056	0.0258	*Weeksella*	0.1222	0.1274	0.7362
*Leptotrichia*	4.2630	1.9563	2.1356				

### Members of the individual oral microbiomes were represented in samples of saline pulling and oil pulling

**Fig. 6:**
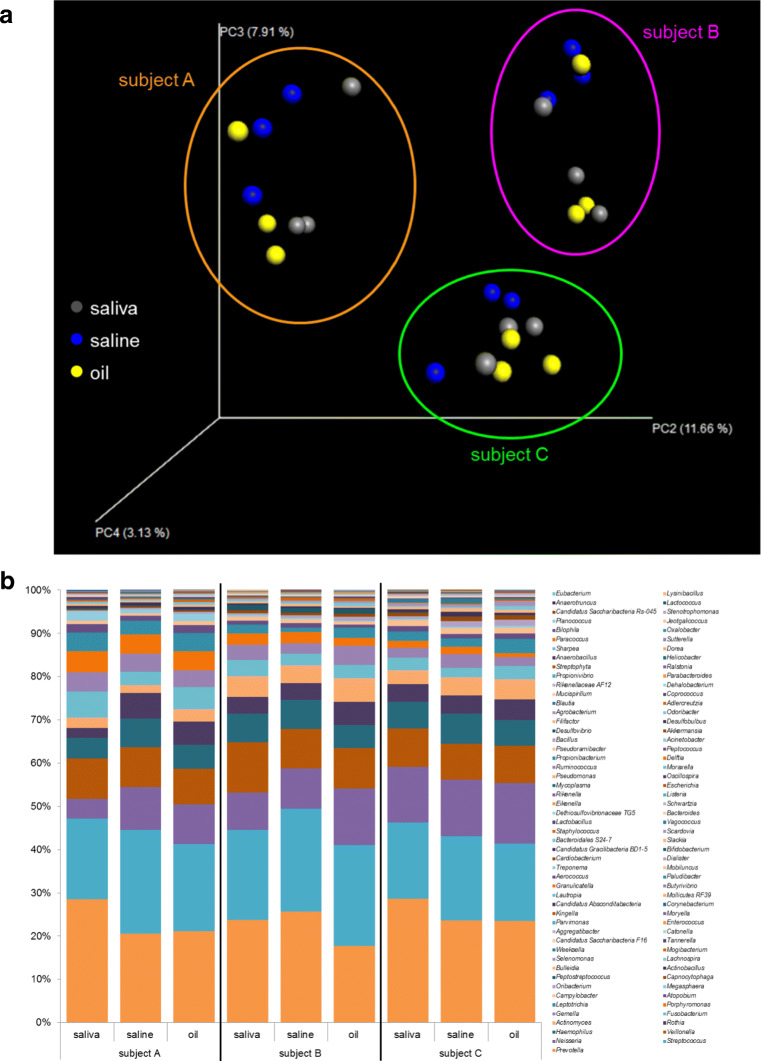
Oral microbiomes of subjects A, B, and C subdivided into saliva, saline pulling, and oil pulling. **a** Principal coordinates analysis (PCoA) of *n* = 9 data points of subjects A, B, and C: saliva (gray) = numbers 01, 12, 22; saline (blue) = numbers 03, 06, 09; oil (yellow) = numbers 13, 16, 19 (Table 1). Data points from each subject appear as distinct cloud, indicated in orange (subject A), purple (subject B), and green (subject C). **b** Percentage of bacteria down to the genus level indicated as columns for subjects A, B, and C subdivided into saliva, saline pulling, and oil pulling

In order to determine the capacity of saline pulling and oil pulling to absorb members of the oral microbiome representatively, saliva samples, saline pulling samples, and oil pulling samples have been compared (Fig. 6). The PCoA in Fig. 6a is the same analysis as in Fig. 5a but only saliva, saline, and oil pulling samples are shown to demonstrate similarities. Figure 6 b shows the percentage of bacteria down to the genus level found in these sample types. The majority of bacteria are similar and only minor differences in percentage of appearance could be observed. For instance, in subject A, *Prevotella* in saline pulling (20.5%) and in oil pulling (21.2%) samples was lower than in saliva (28.5%), whereas *Neisseria* (9.9%/9.2%) and *Rothia* (5.9%/5.3%) were higher in saline/oil pulling samples than in saliva (4.5%/2.3%). In subject B, *Prevotella* was lower in oil pulling samples (17.7%) than in saline pulling samples (25.7%) and in saliva (23.7%). In subject C, *Prevotella* was higher in saliva (28.6%) than in saline (23.6%) and in oil pulling (23.5%) samples.

**Fig. 7:**
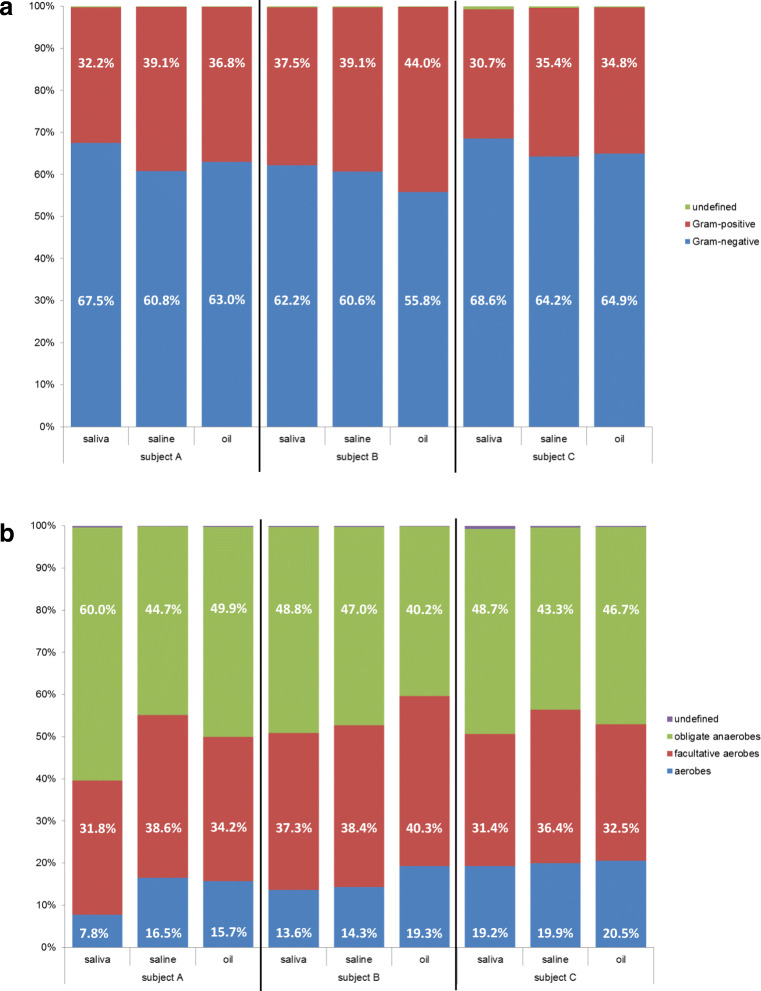
Oral microbiomes of subjects A, B, and C subdivided into saliva, saline pulling, and oil pulling. **a** Subdivision into percentage of Gram-negative and Gram-positive bacteria. **b** Subdivision into percentage of obligate anaerobes, facultative aerobes, and aerobes

Furthermore, bioinformatics was done considering cell wall structure (Gram stain: Gram-positive, Gram-negative) and oxygen tolerance (obligate anaerobes, facultative aerobes, aerobes) as demonstrated in Fig. 7. The ratios of Gram-positive to Gram-negative bacteria were very similar for saline and oil pulling samples but contained less Gram-negative and more Gram-positive bacteria when compared with saliva (Fig. 7a). The ratios of obligate anaerobes, facultative aerobes, and aerobes were very similar, apart from subject A who showed significant enrichment of aerobes in saline and oil pulling samples. Subject B showed a slightly increased enrichment of aerobes in oil pulling samples (Fig. 7b).

### Quality of mock community in saline and in oil

**Fig. 8:**
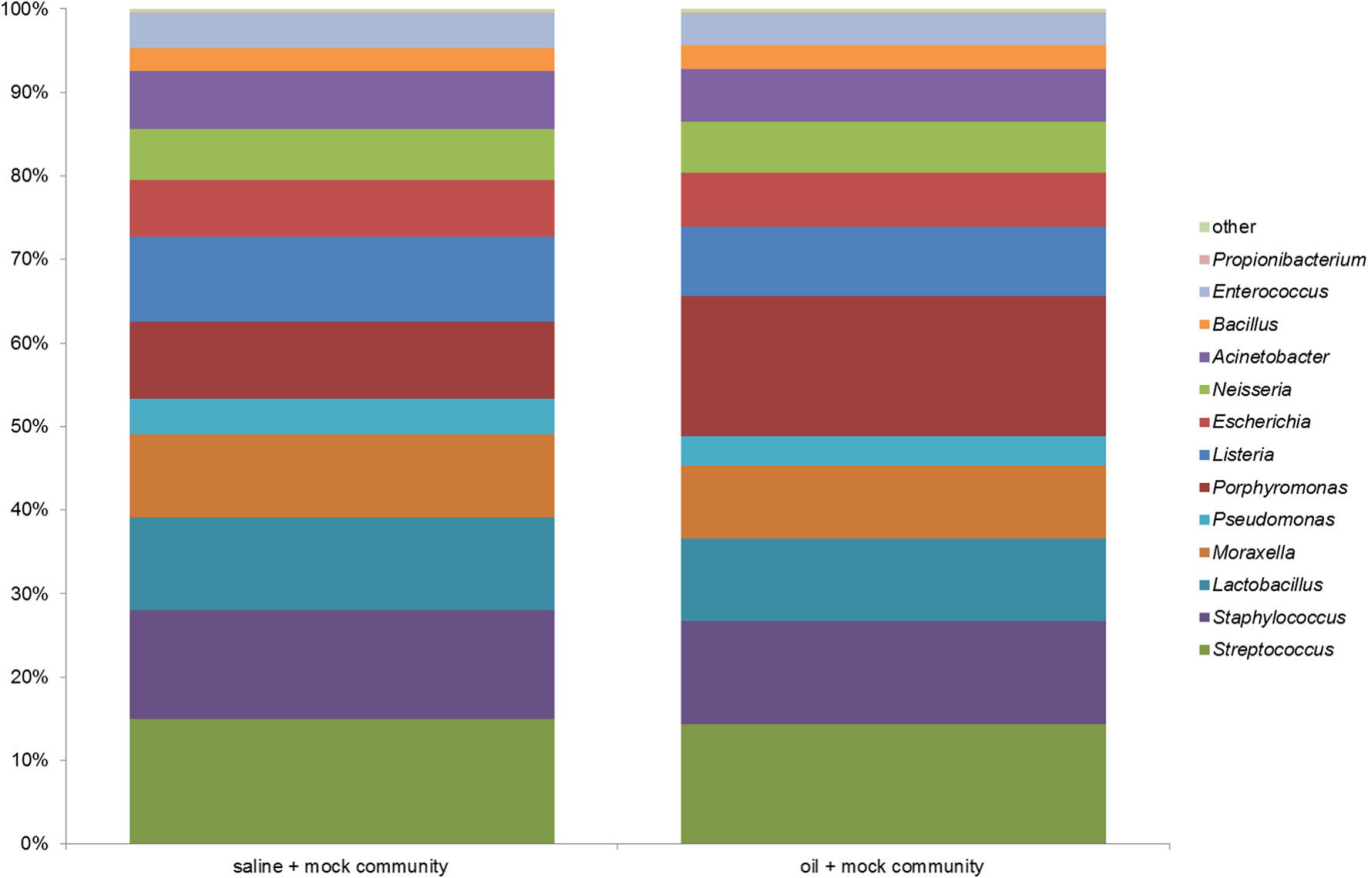
Mock community as quality and positive control for saline pulling (negative control) and for oil pulling as the verum (Table 2)

In order to monitor the whole procedure of DNA extraction, PCR, library construction, and 16S rDNA next-generation sequencing, a mock community was created containing equimolar amounts of 14 Gram-positive and Gram-negative bacteria taking into account morphologies and cell wall structures (Table 1). Four different types with respect to percentage of appearance have been detected (Fig. 8): firstly, enrichment of *Porphyromonas* in oil (saline: ~ 9%, oil: ~ 17%); secondly, percentage of appearance in the range of ~ 10 through ~ 15% in saline and oil (*Streptococcus*, *Staphylococcus*, *Lactobacillus*, *Moraxella*, *Listeria*); thirdly, percentage of appearance in the range of ~ 4 through ~ 7% in saline and oil (*Escherichia*, *Neisseria*, *Acinetobacter*, *Pseudomonas*, *Bacillus*, *Enterococcus*); fourthly, low abundance of *Propionibacterium* (saline: ~ 0.17%, oil: ~ 0.22%).

## Discussion

The human oral cavity is a unique habitat with distinctive anatomic and physiological features resulting in a highly diversified oral environment and microbiota. Individual oral health depends on personal oral hygiene in order to reduce the overall microbial burden, including potential pathogens, and on nutrition. Both, oral hygiene and nutrition, have a significant impact on oral health and the incidence of oral and systemic diseases [35–41]. Apart from teeth brushing, utilization of dental floss, and rinsing with mouthwashes, oil pulling is a very old and cost-effective enlargement to oral and systemic health benefits. This study on oil pulling was conducted with three healthy subjects all part of the laboratory team in order to ensure continuity and immediate sample processing. To our knowledge, this was the first study which examined the microbiota in oil pulling samples. Therefore, we aimed at the high-resolution taxonomic examination of the oral microbiota in saliva and in oil pulling samples and analyzed the capacity of the method to reduce the overall microbial burden of the oral cavity transiently.

The first decision to conduct the study concerned the volume of sunflower seed oil for pulling and duration of procedure. Instructions in literature regarding volume are very imprecise and range from 1 to 3 teaspoons, one tablespoon through as much as comfortable [15–17, 20]. In order to specify volume, we tested 5, 10, 15, 20, and 25 ml and found that 15 ml was acceptable for all participants. Regarding duration, we used 15 ml of oil and tested oil pulling for 10, 15, and 20 min. All participants found 15 min acceptable and reported on an increasing volume during pulling and a growing viscosity which came along with a “peeling” effect in mouth. Both effects, increased volume and viscosity, were considerably less when saline is used instead of oil. Examination of the sample volumes after saline and oil pulling revealed a significant higher production of saliva for the latter one explaining the observation that volume during pulling increased considerably (Fig. 1). Therefore, oil pulling has the capacity to stimulate the salivary flow rate and can be recommended for patients suffering from hyposalivation, xerostomia, or dry mouth syndrome [42, 43]. In order to examine the feeling of an increased viscosity and the accompanying “peeling” effect, we microscoped saline/saliva and oil/saliva samples. The analysis revealed that oral epithelial cells were closely ensheathed with distinct oil droplets in oil pulling samples, whereas the cells in saline pulling samples appeared distinct. The major components of the sunflower seed oil used were oleic acid (14–40%) and linoleic acid (48–74%) which are monounsaturated omega-9 fatty acids and polyunsaturated omega-6 fatty acids, respectively. We assume that the intensive pulling with that oil resulted in a mixture, an emulsion, of oil and saliva which induced generation of micelles interacting via their unsaturated cis double bonds with superficial triglycerides such as lecithin and glycerol of the oral epithelial cells through van der Waals forces. The longer the pulling lasted, the more epithelial cells were ensheathed, inclosing bacteria, and thereby increasing emulsification and viscosity which induced the “peeling” effect. Therefore, oil pulling can be construed as a kind of “oral massage” reaching almost all anatomic features in the oral cavity and which is rejected after the procedure. This may contribute to the explanation of the benefits of oil pulling for gingivitis, periodontitis, and tissue regeneration [16, 18, 19].

16S rDNA sequencing is a complex procedure involving DNA extraction, PCR, library construction, next-generation sequencing, and bioinformatics resulting in a relative frequency of bacteria in a given sample as a function of sequence counts in percentage. In order to calculate the amount of and to identify viable bacteria in saliva, saline pulling, and oil pulling samples, we cultured the samples under appropriate microaerophilic and anaerobic conditions [44]. The advantage of our study was the immediate sample processing which ensured optimal culture conditions resulting in a bacterial survival rate of ~ 90–98% cultivable bacteria within 1 h. Within that time period, oil pulling showed no significant anti-bacterial effect on cultivable bacteria which was in accordance with the results of other studies [18, 20]. Comparison of culture and sequencing showed that we were able to culture members of 12 genera out of 85 genera detected by NGS. Although these 12 genera represented only ~ 14%, these cultivated genera belong to the mass of ~ 80–85% genera of the oral microbiome (Table 4; Fig. 5) indicating a reliable counting of viable bacteria in saliva, saline pulling, and oil pulling samples in order to calculate the reductive effect of pulling on the microbial burden in oral cavity. Seven more bacteria were cultivated but not detected by NGS. One explanation is that the colony-forming units of *Achromobacter*, *Leifsonia*, *Leuconostoc*, *Micrococcus*, and *Nocardia* were very low and therefore not detected by NGS. The second explanation is that the genera *Klebsiella* and *Serratia* belong to the *Enterobacteriaceae*. Taking into account that the analyzed amplicon had a size of ~ 250 bp, we assume that these bacteria were false-classified by the bioinformatics software tool to other *Enterobacteriaceae* (Table 4).

*Candida albicans* was the only fungus cultivated and which was not covered by 16S rDNA NGS since the method used focused on bacteria. But our study showed that oil pulling had also the capacity to reduce the amount of this pathogen significantly (Fig. 4b). The overall reduction of the microbial burden in the oral cavity due to oil pulling was transient since the same amount of microorganisms was restored within ~ 24 h. Furthermore, the high-resolution taxonomic examination of the oral microbiome exhibited that the individuals who participated in the study harbored individual oral microbiomes and that the same composition of microorganisms was also restored after ~ 24 h. This was consistent with the oral cavity as a highly diversified oral environment but harboring a core microbiome and maintaining individuality with little geographic diversity [3, 5, 45, 46].

Even more, the individual microbiomes were detected in all samples examined independently whether in saliva, saline, or oil pulling samples (Figs. 5 and 6). The pulling methods showed no significant preferences for particular bacteria, even considering morphology, cell wall structure, and oxygen tolerance (Fig. 7). Therefore, the overall microbial burden was uniformly reduced. But one has to bear in mind that the participating subjects were healthy, nonsmoker, and omnivore and showed no current oral health problems. All participants applied in general a very good oral hygiene. Considering the mock community used as a positive control for the whole analysis, we observed a significant enrichment of the well-known oral pathogen *P. gingivalis* in oil pulling sample (Fig. 8). Therefore, it is recommended to conduct a study with patients suffering from periodontitis where *P. gingivalis* plays a crucial role and acts as a keystone-pathogen [47, 48] in order to demonstrate potential benefits of oil pulling for periodontitis via reduction of this pathogen in the oral cavity.

The participants (*n* = 3) of this study perceived the oil pulling procedure as comfortable and reported no problems, which is in compliance with other studies [15–22]. However, literatures report on at least three cases where parts of the oil/saliva emulsion were aspirated, inhaled, or swallowed thereby causing exogenous lipoid pneumonia [49, 50]. This has to be considered and requires detailed instructions for participants and patients prior to oil pulling.

## Conclusions

To sum up, this was the first comprehensive study which examined the microbiota in oil pulling samples. The data achieved within the limitations of this pilot study show that oil pulling is able to reduce the overall microbial burden of the oral cavity transiently and that the microbiota in oil pulling samples represent the entire oral microbiome. As an enlargement of standard oral hygiene techniques, the combination of oil pulling, e.g., along with teeth brushing, has the capacity to minimize the risk of local and distant-site infections contributing to overall oral health. Although this is conclusive and considering publications on oil pulling, to our opinion evidence necessitates clinical trials (based on the standardized and commercially available sunflower seed oil we used, a particular volume of 15 ml and pulling for exactly 15 min) with sufficient participants and takes additional questions into account: (1) Do oil pulling samples contain other substances apart from microorganisms, for example, bacterial proteins, toxins, or any other molecules? This could be answered by using mass spectrometry as a direct analysis. (2) Has oil pulling the capacity to minimize chronic inflammation starting from oral cavity? This could be answered by examination of specific parameters for inflammation such as CRP, leukocyte amount, immunoglobulins, erythrocyte sedimentation rate, and complete blood count in blood samples as an indirect analysis.
